# Loss of Chromosome Y Associates With Altered Immune Cell Trajectories and X‐Inactivation Features

**DOI:** 10.1111/acel.70528

**Published:** 2026-05-03

**Authors:** Ahmed Dawoud, Luke Green, Owen Rackham

**Affiliations:** ^1^ School of Biological Sciences University of Southampton Southampton UK; ^2^ Faculty of Medicine University of Southampton Southampton UK

## Abstract

The loss of chromosome Y (LOY) in leukocytes is the most prevalent form of clonal mosaicism observed in older men. Previous studies provided multiple pieces of evidence for the effect of LOY on the immune system and connected LOY to elevated risk of all major causes of mortality, including cardiovascular diseases and cancer. Despite these associations, the dynamic effects of LOY across the developmental trajectories of immune cell populations remain unclear. We utilized single‐cell RNA‐sequencing data from the peripheral blood mononuclear cells of 416 male donors (median age = 68) from the OneK1K cohort. LOY was identified in 45,304 cells (8.76%) and exhibited cell type–specific effects on immune cells along the differentiation trajectories. The largest frequency was detected in monocytes (18.6% in classical and 17.1% in nonclassical) with a progressive decrease along the transition trajectory from 22.6% to 15.8% (*p*_adj_ = 2.00 × 10^−11^), and a gradual reduction in the expression of nonclassical markers *LYPD2* and *C1QA*. LOY is associated with a profibrotic signature in classical monocytes marked by downregulation of *IL1B* (log FC = −0.22, *p*_fdr_ = 2.84 × 10^−6^) and MYC‐regulated genes (log FC = −0.25, *p*_fdr_ = 2.22 × 10^−5^), consistent with previous observations that LOY‐associated macrophages are polarized toward a fibrotic rather than inflammatory phenotype in cardiac and pulmonary injury. Notably, we detected aberrant expression of *XIST*, the essential X‐chromosome‐inactivation lncRNA that is not normally expressed in males, and upregulation of genes known to escape X‐inactivation, including male‐biased cancer‐related genes *KDM6A*, *DDX3X*, *KDM5C*, and *ZRSR2*. Our results indicate associations between LOY and cell type–specific transcriptional changes, including aberrant X‐inactivation features.

## Graphical abstract

Loss of chromosome Y in male leukocytes exhibits cell type‐specific effects on immune cell phenotypes and transcriptional profiles along differentiation trajectories. LOY is associated with profibrotic monocyte signatures and aberrant X‐inactivation gene expression, highlighting links to immune dysfunction and age‐related diseases in men.

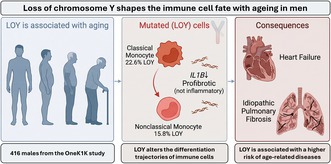

## 1 Introduction

Human hematopoiesis is the efficient, yet age‐dependent, process of generating blood and immune cells. This process is challenged by the acquisition of somatic point mutations (Genovese et al. 2014; Jaiswal et al. 2014) and/or large chromosomal alterations (Jacobs et al. 2012; Laurie et al. 2012) over time. These mutations can provide a selective growth advantage and drive clonal expansion in blood (Steensma et al. 2015). Among all detected somatic mutations, the loss of chromosome Y (LOY) in the peripheral blood of men is the most prevalent event (Forsberg et al. 2014; Zhou et al. 2016) that affects more than half of men by the age of 80 years (Francis et al. 2024; Thompson et al. 2019). The Y chromosome is the smallest human chromosome and plays a key role in male sex determination, hosting genes essential for spermatogenesis (Berta et al. 1990). However, large population studies indicated a wider role for the Y chromosome in the progression of hematological malignancies (Baliakas and Forsberg 2021), solid cancers (Forsberg et al. 2014), nonmalignant diseases such as Alzheimer's disease (Dumanski et al. 2016), and cardiovascular diseases (Haitjema et al. 2017; Sano et al. 2022), and have linked LOY to altered levels of serum biomarkers (Dawoud et al. 2023; Stankovic et al. 2021).

One common feature of these disease associations is the effect of LOY on the immune system, either at the population scale or in specific disease contexts. For example, LOY has been associated with an increase in leukocytes in the Biobank Japan cohort (Terao et al. 2019) and, more specifically, neutrophils and monocytes in the UK Biobank cohort (Lin et al. 2020). An association between LOY and an increase in Natural killer (NK) cells was observed in Alzheimer's disease (Dumanski et al. 2021), while LOY led to an increase in CD4^+^ T cells in prostate cancer (Dumanski et al. 2021) and pulmonary fibrosis (Wang et al. 2024). Beyond these associations, LOY has also been shown to alter immune cell fates. LOY promoted a bias toward regulatory‐T‐cell‐fate within the CD4^+^ population in peripheral blood and tissues (Mattisson et al. 2024). Similarly, LOY was shown to promote a bias toward fibrotic rather than inflammatory phenotypes in monocyte‐derived cardiac (Sano et al. 2022) and alveolar (Wang et al. 2024) macrophages. Collectively, these associations between LOY and the immune system highlight the potential value of LOY‐associated markers as novel therapeutic targets and as risk predictors for age‐related diseases (Xu et al. 2023).

Genome‐wide association studies (GWAS) have revealed the genetic risk factors of LOY in healthy individuals (Terao et al. 2019; Thompson et al. 2019; Wright et al. 2017; Zhou et al. 2016). The most prominent marker was rs2887399 located in the promoter region of *TCL1A*, a marker for B lymphocytes, where the T allele was associated with a lower risk of LOY (Zhou et al. 2016). Single‐cell RNA‐sequencing (scRNA‐seq) technology has allowed the exploration of cell type–specific expression of the markers identified by GWAS (Dumanski et al. 2021). *TCL1A* was upregulated 1.75‐fold in B lymphocytes with LOY in comparison to non‐LOY cells (Thompson et al. 2019). Furthermore, differential gene expression analysis of scRNA‐seq data identified autosomal and X‐linked genes associated with LOY (Dumanski et al. 2021; Mattisson et al. 2021, 2024). These genes were enriched in pathways related to RNA processing, immune response, and cellular proliferation (Dumanski et al. 2021). Further exploration of larger single‐cell datasets would help to characterize the effect of these associations on cell fate.

OneK1K (Yazar et al. 2022), part of Phase 1 of the Tenk10k, is an initiative that performed genotyping and transcriptional profiling of the peripheral blood mononuclear cells (PBMCs) of 982 individuals to identify genetic risk factors associated with gene expression at the cell type level. In this study, we aimed to characterize the prevalence of LOY across major immune cell types and along their lineage developmental trajectories in individuals from OneK1K. Furthermore, we sought to identify autosomal and X‐linked genes associated with LOY that affect immune cell fate and represent a potential therapeutic and diagnostic target for age‐related diseases.

## 2 Results

### 2.1 Prevalence of LOY Across Different Immune Cell Types

Recent advances in the multiplexing of scRNA‐seq to handle cells derived from 100s of donors in a single experiment (Shapiro et al. 2013) have powered a new range of applications, including longitudinal studies and genotype‐expression associations (van der Wijst et al. 2018). We analyzed the data from two independent methods: scRNA‐seq of PBMCs and SNP arrays of whole blood DNA to call LOY in 416 males aged between 19 and 93 years (median = 68) within the OneK1K cohort.

**FIGURE 1:**
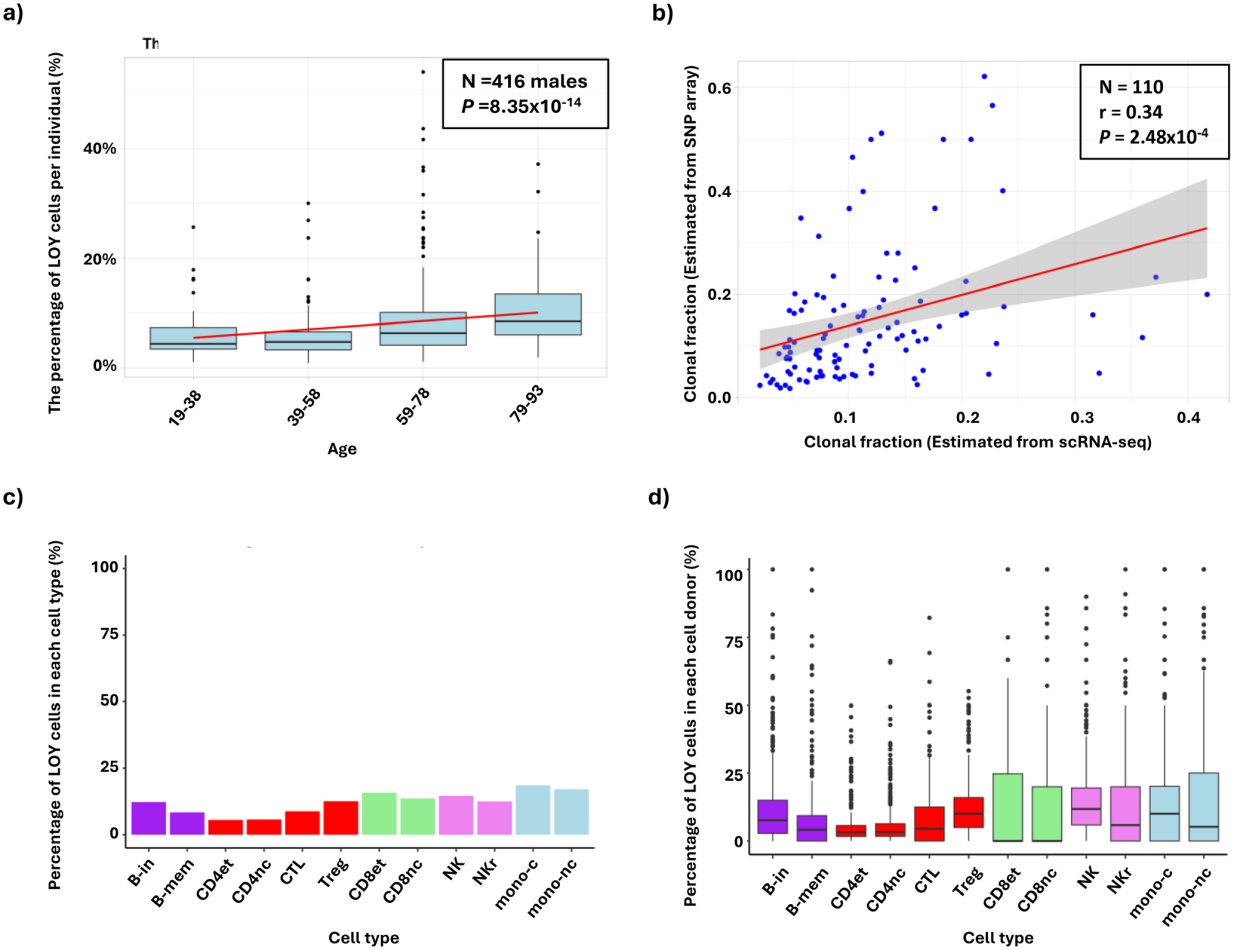
The prevalence of LOY in scRNA‐seq and SNP arrays of 416 males in OneK1K data. (a) Box plot illustrating the relationship between age, divided into four bins (19–38, 39–58, 59–78, and ≥ 79), and the percentage of LOY cells per individual. LOY was defined by the absence of expression counts of male‐specific Y chromosome genes, derived from the combined Cell Ranger and Velocyto methods. The red line represents the linear regression fit. (b) Scatter plot showing the correlation between clonal fraction estimates from SNP arrays and single‐cell data. Each dot represents an individual, and Pearson correlation coefficient (*r*) and *p* value are shown on the panel. For visualization, the red line represents the linear regression fit with 95% confidence interval. (c) Bar plot showing the average percentage of LOY cells identified in each cell type. Percentages were calculated per cell type as (number of LOY cells/total cells in that type) × 100. *n* (total cells) for each cell type. (d) Box plot displaying the percentage of LOY cells per donor across different cell types. Dots indicate outliers.

For calling LOY in scRNA‐seq, we followed the previously published method (Mattisson et al. 2024); LOY identity was allocated to a cell if no reads were assigned to the male‐specific region (MSY) on the Y chromosome using two methods for read counting: cell ranger (Zheng et al. 2017) and velocyto (La Manno et al. 2018). From a total of 517,412 cells, we identified 45,304 cells (8.76%) in 416 male donors with LOY (Table S1). The prevalence of LOY cells at the individual level ranged from between 1% (*n* = 10/1123 cells) and 54% (*n* = 1416/2621 cells) per donor. Expanded clonality, which we defined by the observation of LOY in 10% or more of cells, was assigned to 25.7% (*n* = 107 donors, median age = 71). Adjusting for the effect of sequencing pool and other major sources of variation (see Section 4), the prevalence of LOY cells was significantly associated with age (*β* = 0.013; 95% CI, 0.009–0.016; *p* = 8.35 × 10^−14^) (Figure 1a). To assess robustness to sequencing depth, we repeated the donor‐level age analysis after applying per‐cell UMI sensitivity filters that removed the lowest 5%, 10%, and 15% of cells. In all cases the age effect remained highly significant, and the point estimate changed by < 1% (5% filter: *β* = 0.015; 0.011–0.019; *p* = 2.74 × 10^−14^), (10% filter: *β* = 0.016; 0.012–0.019; *p* = 4.40 × 10^−14^), (15% filter: *β* = 0.017; 0.013–0.021; *p* = 4.37 × 10^−15^). These results indicate that the observed association with age is not driven by low‐coverage cells.

For calling LOY in SNP‐array data, we used the phased‐genotypes segmentation method (Loh et al. 2018) and identified LOY in the leukocytes of 26.4% (*n* = 110) of males, with clonal fractions ranging from 0.02 to 0.62, and expanded clonality in 58 males (13.9%). Notably, autosomal mosaic chromosomal alterations (mCA) were identified from SNP array data in 26 donors (28 events in total, Table S1). Of them, seven donors have both LOY and autosomal mCA. We did not observe a significant association between mCA and LOY (*χ* ^2^ test, *p* = 0.9). In this study, we focus on LOY specifically, as autosomal mCAs are substantially more challenging to detect robustly in scRNA‐seq data, whereas LOY can be inferred with higher confidence due to its clear gene dosage signature in the Male‐Specific Region (MSY) of the Y chromosome.

One important feature of PBMCs processed for scRNA‐seq, in contrast to whole blood samples used for SNP arrays (Hoffbrand et al. 2024), is that they only contain agranulocytes (monocytes and lymphocytes) and lack granulocytes (neutrophils, basophils, and eosinophils). However, genotyping data generated by SNP arrays and DNA extracted from whole blood samples are commonly used to assess acquired chromosomal mosaicism with a sensitivity up to 1% (Loh et al. 2018; Thompson et al. 2019). We hypothesized that a significant proportion of LOY events with overrepresentation in agranulocytes could be missed when we use DNA genotyping methods. To investigate this hypothesis, we assessed the relationship between the frequency of LOY per donor between scRNA‐seq calls and SNP arrays calls. LOY identified by scRNA‐seq has weak linear correlation with the clonal fraction detected by SNP arrays (*r* = 0.34; 95% CI, 0.17–0.50; *p* = 2.48 × 10^−4^, Figure 1b). Furthermore, all males with expanded clonality, defined by a clonal percentage of 10% or more in SNP arrays data, have been detected using scRNA‐seq with 4.7% or more of LOY in PBMC cells; however, only 50.5% (*n* = 54/107) of expanded clonality defined by scRNA‐seq calls were defined as LOY by SNP arrays. This observation suggests that SNP arrays failed to detect LOY events in leukocytes of donors with a high representation of LOY in the agranulocyte compartment. This highlights the potential of scRNA‐seq to provide new insights into LOY, surpassing the limitations of genotyping‐based methods.

To assess the distribution of LOY across major cell types; monocytes, B cells, Natural Killer cells, CD4^+^ T cells, and CD8^+^ T cells, we classified cells using a scheme derived from Yazar et al. (2022). In brief, we group cells into one of the five major cell lines after excluding minor cell types. Each major cell type was analyzed independently, and cells were clustered and annotated according to the scheme in Table 1.

**TABLE 1:** Major cell types analyzed for the prevalence of LOY across 416 donors.

Immune cell type^a^, ^b^, ^c^, ^d^	Abbreviation	Cell markers^g^	Number of clusters^e^	LOY cells, *n* (%)	Total cells, *n*
CD4^+^ naïve and central memory T cell	CD4nc	*CD3D*, *CD4*, *CCR7*, *SELL*, *LRRN3*	7	4431 (5.8)	76,835
CD4^+^ effector memory and central memory T cell	CD4et^f^	*CD3D*, *CD4*, *KLRB1*, *GZMK*, *TNFSF13B*, *IL7R*	2	3811 (5.6)	67,859
Regulatory T cells	Treg	*CD3D*, *FOXP3*	1	1335 (12.57)	10,615
Cytotoxic T lymphocytes	CTL^f^	*CD3D*, *KLRB1*, *ID2*	1	691 (8.8)	7880
CD8^+^ naïve and central memory T cell	CD8nc	*CD3D*, *CD8A*, *LTB*, *CCR7*, *PASK*	2	246 (13.7)	1809
CD8^+^ effector memory T cell	CD8et	*CD3D*, *CD8A*, *GNLY*, *NKG7*, *KLRB1*	1	358 (15.8)	2265
Natural killer cell	NK	*CD56*, *GZMA*, *GZMB*	2	3033 (14.6)	20,750
Natural killer cell recruiting	NKr	*CD56*, *GZMK*, *XCL1*, *XCL2*	1	546 (12.5)	4366
Immature and naïve B cell	B‐in	*CD20*, *TCL1A*, *FCER2*, *IL4R*	4	2475 (12.3)	20,160
Memory B cell	B‐mem	*CD20*, *CD27*, *TNFRSF13B*	2	1098 (8.4)	13,126
Classical monocyte	Mono‐c	*CD14*, *LYZhi*	3	1777 (18.6)	9561
Non classical monocyte	Mono‐nc	*CD16*	2	673 (17.1)	3927

^a^ CD34^+^ cells were excluded for all groups.

^b^ CD8A/B cells were excluded for CD4 T cells analysis.

^c^ CD4, CD3G/D/E cells were excluded CD8 T cells analysis.

^d^ CD4, CD3G/D/E, and CD8A/B cells were excluded for B cells, monocytes, and NKs.

^e^ Number of clusters identified by shared nearest neighbor method.

^f^ For the purpose of building the differentiation trajectory of CD4^+^ T cells to Treg cells, uninformative clusters of CTL and CD4et had been removed. A total of 55,424 CD4et cells were retained for building the trajectory.

^g^ Cell markers are listed as they were used in OneK1K (Yazar et al. 2022).

Previous studies have shown the highest frequency of LOY in monocytes and NK (Dumanski et al. 2021; Mattisson et al. 2021, 2024). We refined this observation into defining classical monocytes (mono‐c) to have the highest frequency (18.6%) (see Figure 1c), and defining NK with the highest median of LOY per donor (11.76%, IQR = 13.6), followed by Treg (10%, IQR = 11) and mono‐c (10%, IQR = 20.2, Figure 1d). The relationship between single‐cell calls and SNP array calls varies across cell types (Table S2 and Figure S1). The clonal fraction estimated from SNP arrays has a strong linear relationship with those estimated from scRNA‐seq calls in monocyte subclasses: classical monocytes (mono‐c, *r* = 0.75; 95% CI, 0.61–0.84; *p*_fdr_ = 2.20 × 10^−11^), and nonclassical monocytes (mono‐nc, *r* = 0.66; 95% CI 0.44–0.80; *p*_fdr_ = 1.04 × 10^−5^). Moderate relationships were detected between the clonal fraction estimated from SNP arrays and those estimated from NK cells (NK, *r* = 0.51; 95% CI, 0.35–0.64; *p*_fdr_ = 1.88 × 10^−7^), and NK recruiting cells (NKr, *r* = 0.57; 95% CI, 0.33–0.74; *p*_fdr_ = 1.26 × 10^−4^), and weak correlation was identified with one subset of B cells; immature and naïve cells (B‐in, *r* = 0.31; 95% CI 0.12–0.47; *p*_fdr_ = 4.59 × 10^−3^), but no relationship was found with memory B cells (B‐mem). Furthermore, we found no correlation between the clonal fraction estimated by SNP arrays and any of the CD4 or CD8 T cell subtypes (i.e., naive and central memory [CD4nc], effector [CD4et], cytotoxic [CTL], and regulatory cells [Treg], naive and central memory [CD8nc], and effector memory [CD8et]).

These observations highlight the bias of LOY calls derived from SNP arrays data toward representing the myeloid compartment of immune cells, whereas LOY calls derived from scRNA‐seq data appear capable of assessing both the myeloid and lymphoid compartments. In the following sections, we dissect the effect of LOY defined by scRNA‐seq on the phenotype and transcriptional profiles of major immune cell types.

### 2.2 The Effect of LOY on the Fate of Immune Cells

**FIGURE 2:**
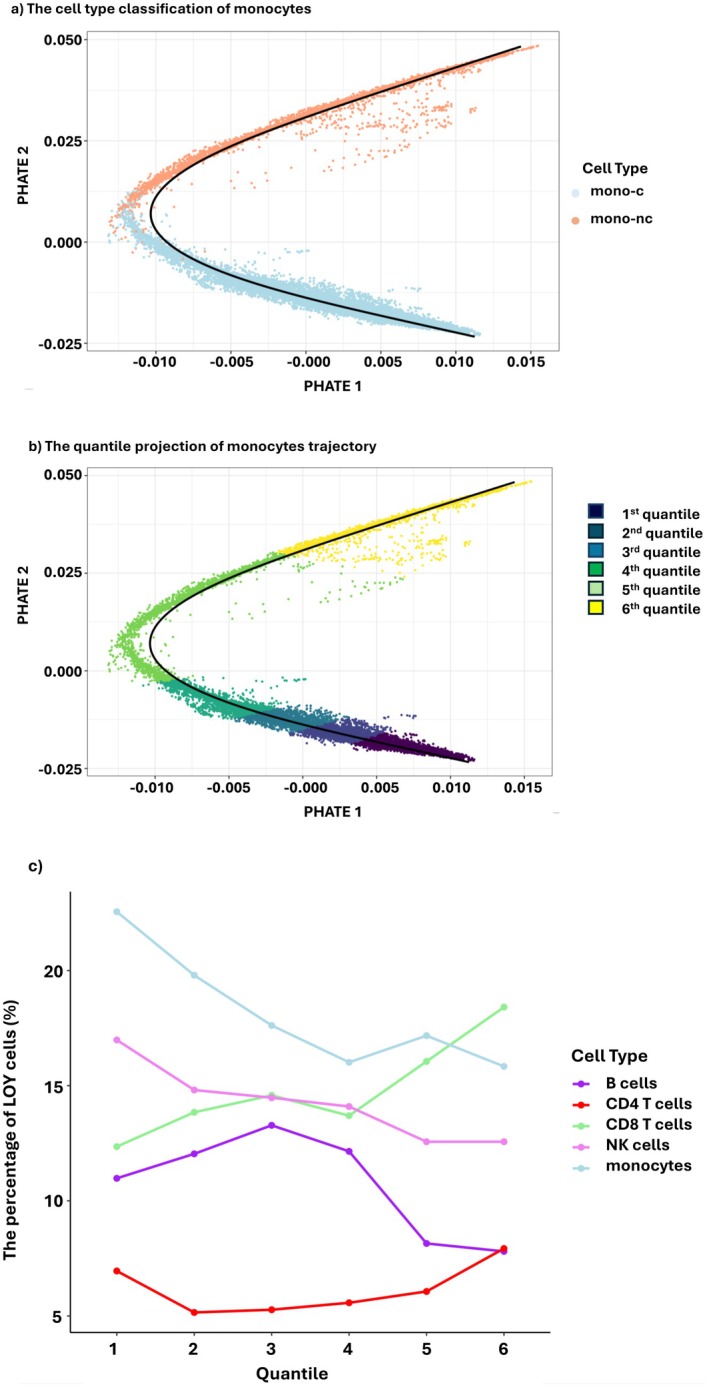
The percentage of LOY across cell trajectories. Cells were categorized into five major cell types and further classified along their developmental trajectory using pseudotime analysis and six equal quantiles, where Q1 represents the earliest developmental state and Q6 represents the latest stage. (a) Cell type projections visualized using PHATE dimensionality reduction and the transition from classical monocytes to nonclassical monocytes. (b) Quantile projections visualized using PHATE dimensionality reduction from classical monocytes to nonclassical monocytes. (c) Line plot showing the relationship between the percentage of LOY cells and pseudotime quantiles across different cell types.

Two independent studies have identified a lineage bias associated with LOY in CD4^+^ T cells, marked by an overrepresentation of LOY cells in T regulatory (Treg) cell populations within both peripheral blood and cancer tissues (Mattisson et al. 2024; Wójcik et al. 2024). To further investigate potential lineage biases across major cell types, we assessed the relationship between LOY frequency and cell classification using two approaches: (i) by marker‐defined subtypes (Figures 2a) and (ii) by the cell position on a cell type developmental trajectory. These trajectories capture critical aspects of cell type flux (i.e., immature and naïve B cell transition to memory B cell) and were stratified into six equal quantiles based on pseudotime along the trajectory (Figure 2b). To evaluate whether the observed trajectory association could be explained by age‐related compositional shifts in the dataset, we performed a sensitivity analysis stratifying donors by the median age (68 years).

Initially, we confirmed the previous observation that LOY cells are enriched for Treg cells (*n* = 1335/10,615; 12.57%) compared with other CD4^+^ T cells (*n* = 8933/152,574; 5.8%). Other CD4^+^ T cells are composed of CD4et (*n* = 3811/67,859) 5.6%, CD4nc (*n* = 4431/76,835) 5.8%, and CTL (*n* = 691/7880) 8.8%, and using a multivariate model adjusted for age, and first 10 genotypic principle components (PCs) (OR = 2.41, *p* = 3.42 × 10^−157^) (Table S3). Next, we constructed the developmental trajectory of CD4^+^ T cells transitioning toward a Treg state (Figure S1a). The developmental trajectory of Treg cells represents their progression from uncommitted naive cells (Zhu et al. 2009). Notably, effector T cells can acquire regulatory functions under specific conditions, transitioning into Treg cells, marked by the expression of *FOXP3* (Bluestone and Abbas 2003). We used PHATE dimensionality reduction to visualize the CD4^+^ T to T‐reg manifold, and to construct its developmental trajectory (see Section 4 for details). This allowed us to detect the expected significant positive association between LOY (OR = 1.04, *p*_adj_ = 8.89 × 10^−8^) and the positioning of cells along the developmental trajectory (Figure S1b,c). In the age‐stratified analysis, the positive association remained consistent in both younger (< 68 years, OR = 1.03, *p* = 6.26 × 10^−3^) and older (≥ 68 years, OR = 1.05, *p* = 1.63 × 10^−6^) donor groups, with comparable effect sizes (Table S4). While statistically significant, the magnitude of this effect is relatively modest to other trajectory effects.

Interestingly, CD8^+^ T cells showed a gradual increase in LOY cells across the trajectory of differentiation (Figure S1c,d) from CD8nc (246/1809, 13.7%) to CD8et (358/2265, 15.8%) and from 12.4% in the first quantile to a maximum of 18.4% in the sixth quantile (OR = 1.08, *p*_adj_ = 5.07 × 10^−3^).

In contrast, NK cells, monocytes, and B cells showed a gradual decrease in the percentage of LOY cells across their developmental trajectories. NK cells undergo transformation along their development trajectory toward an activated state (Figure S1e). In the activated state, the cells express chemokines such as *XCL1/2* to recruit other immune cells (Yang et al. 2019). We referred to this state as NK recruiting (NKr) cells. LOY was less prevalent in NKr cells (546/4366, 12.5%) in comparison to other NK cells (3033/20,750, 14.6%, OR = 0.85, *p*_adj_ = 2.51 × 10^−3^) with a consistent decrease across the development trajectory from 17% in the first quartile to 12.6% in the sixth quantile (Figure S1f).

Monocytes are the sole agranulocyte among myeloid cell types. An increased monocyte count is a characteristic feature of LOY (Lin et al. 2020). CD14^+^ CD16^−^ monocytes, known as classical monocytes (mono‐c), represent 85% of circulating monocytes derived from the bone marrow (Patel et al. 2017). They play an essential role in inflammation by migrating to tissues and differentiating into macrophages or dendritic cells. In the circulating blood, classical monocytes transform into CD14^+^ CD16^+^ intermediate and CD14^−^ CD16^+^ nonclassical monocytes (mono‐nc). Initially, we detected a higher representation of LOY in mono‐c (*n* = 1777/9561, 18.6%), against mono‐nc (*n* = 673/3927, 17.1%, OR = 0.87, *p*_adj_ = 0.02) as shown in Table 1 and Table S3. To investigate this further, we constructed a monocyte developmental trajectory capturing the transition from mono‐c to mono‐nc (Figure 2a) and found the association to be consistent across the trajectory of monocytes (Figure 2b) and ranges between 22.6% in the first quantile to 15.8% in the sixth quantile (OR = 0.91, *p*_adj_ = 2.00 × 10^−11^). In the age‐stratified analysis, this association remained consistent in both younger (< 68 years, OR = 0.86, *p* = 4.97 × 10^−10^) and older (≥ 68 years, OR = 0.93, *p* = 8.70 × 10^−5^) donor groups, with comparable effect sizes (Table S4). This gradual decrease is remarkable given the short lifespan of monocytes, which ranges from 1 to 7 days (Patel et al. 2017).

B cells are components of the adaptive immune system, in which immature and naive B cells (B‐in) differentiate into plasma cells or memory B cells upon antigen activation (Morgan and Tergaonkar 2022). B cells produce antibodies of different immunoglobulin isotypes, whereas B‐mem represent the secondary immune responses upon re‐encountering the same antigen (Hoffman et al. 2016). CD20 is a B cell marker that is downregulated upon differentiation to plasma cells. In our analysis, we restricted B cells to those in CD20^+^ clusters, which we further classified into B‐in or B‐mem as outlined above, using markers including *TCL1A* for B‐in and *CD27* for B‐mem (Figure S1g). Here, we observed a decrease in LOY cells from (*n* = 2475/20,160, 12.3%) in B‐in to (*n* = 1098/13,126, 8.4%, OR = 0.63, *p*_adj_ = 7.32 × 10^−20^) in B‐mem. Furthermore, we identified a gradual decrease in LOY cells on the transition trajectory (Figure S1h) from B‐in to B‐mem (OR = 0.92, *p*_adj_ = 8.21 × 10^−12^).

*TCL1A* is a marker for B‐in that is gradually downregulated during the transformation to B‐mem (Wen et al. 2020). The SNP rs2887399 is located upstream of *TCL1A*, and the G allele is associated with both the higher risk of LOY (Zhou et al. 2016) and the upregulation of *TCL1A* in blood (Võsa et al. 2021). Thompson et al. (2019) have previously observed an overexpression of *TCL1A* in B cells with LOY in comparison to non‐LOY cells. Accordingly, including rs2887399 as a covariate in the models assessing the relationship between LOY and B cell subtypes eliminated this association (*p*_adj_ = 0.69), as did the analysis based on pseudotime quantiles (*p*_adj_ = 0.67) (Table S3). Additionally, to ensure that this observation was not a compositional enrichment of LOY in a *TCL1A*‐high compartment, we did not observe a significant difference (average log FC = 0.35, *p*_adj_ = 1) in the expression of *TCL1A* in B‐in with Y chromosome against B‐in without Y chromosome. Here, our observation that LOY is overrepresented in B‐in compared with B‐mem provides a phenotypic explanation for the upregulation of *TCL1A* in B cells with LOY.

Building on this, we identified other known risk factors associated with LOY and assessed their connection with B cells; we found that the G allele of rs10849448, upstream of *LTBR*, was significantly associated with higher risk of LOY (Thompson et al. 2019). By exploring the eQTLGen database of cis‐eQTLs (Võsa et al. 2021), we found that the G allele is associated with downregulation of *LTBR* (G allele, *z* = −29.8, *p* = 2.91 × 10^−195^), and interestingly, the nearby *CD27*, a known marker for B‐mem cells (G allele, *z* = −5.4, *p* = 7.1 × 10^−8^) (Võsa et al. 2021). Because *CD27* is a well‐established marker of B‐mem, this pattern suggests that although the variant is located near *LTBR*, its functional effect may extend to regulating *CD27* and is therefore implicated in the relationship between B cell phenotype and LOY. Our results collectively show that LOY is associated with bias toward B‐in cells marked by the overexpression of *TCL1A*, as opposed to CD27^+^ B‐mem that lack *TCL1A*.

### 2.3 Differential Gene Expression

To capture the expression changes in autosomal and X‐chromosome genes associated with LOY, we assessed the differential gene expression between LOY cells and non‐LOY cells in each cell type (Table S5). In addition, we applied a proportion‐based pseudobulk approach for sensitivity analysis, aggregating LOY cell counts per donor within each cell type (Table S6).

#### 2.3.1 Genes Encoding Cell Surface Immunoproteins

Previous studies have highlighted a downregulation of the surface protein *CD99* in LOY cells (Mattisson et al. 2021), we confirmed this observation in the majority of cell subtypes with the strongest effect in Treg (log_2_ FC = −0.67, *p*_adj_ = 1.15 × 10^−31^) and confirmed this by proportional pseudobulk analysis (effect size = −0.19, *p*_adj_ = 2.21 × 10^−18^), no effect in CD8nc, but with opposite direction in CTL (log_2_ FC = 0.17, *p*_adj_ = 8.37 × 10^−26^). However, the proportion‐based pseudobulk sensitivity analysis (Table S6) did not identify a significant difference in CTL between LOY and non‐LOY. *CD99* is located in the pseudoautosomal region 1 (PAR1) of chromosome X and Y, where we would expect downregulation in all cells with LOY, this CTL‐specific divergence suggests that the impact of LOY on *CD99* expression may be context‐dependent, potentially reflecting the distinct activation and cytotoxic state of CTLs, in which *CD99* has been implicated in regulating cytotoxic effector functions (Zhang et al. 2026).

Although *CD99* is a Pseudoautosomal region (PAR) gene, we observed the downregulation of other non‐PAR cell surface markers: *CD48*, located on chr 1 in all CD4^+^ T cell subtypes, and *CD40LG*, a gene located on the non‐PAR of the X chromosome, in Treg and CD4et cells. Recently, reduced expression of *CD48* was detected in T cell lymphoma and presented as a causal factor for evading NK‐cell‐dependent immunity (Chiba et al. 2022).

#### 2.3.2 Genes Located on the X Chromosome

**FIGURE 3:**
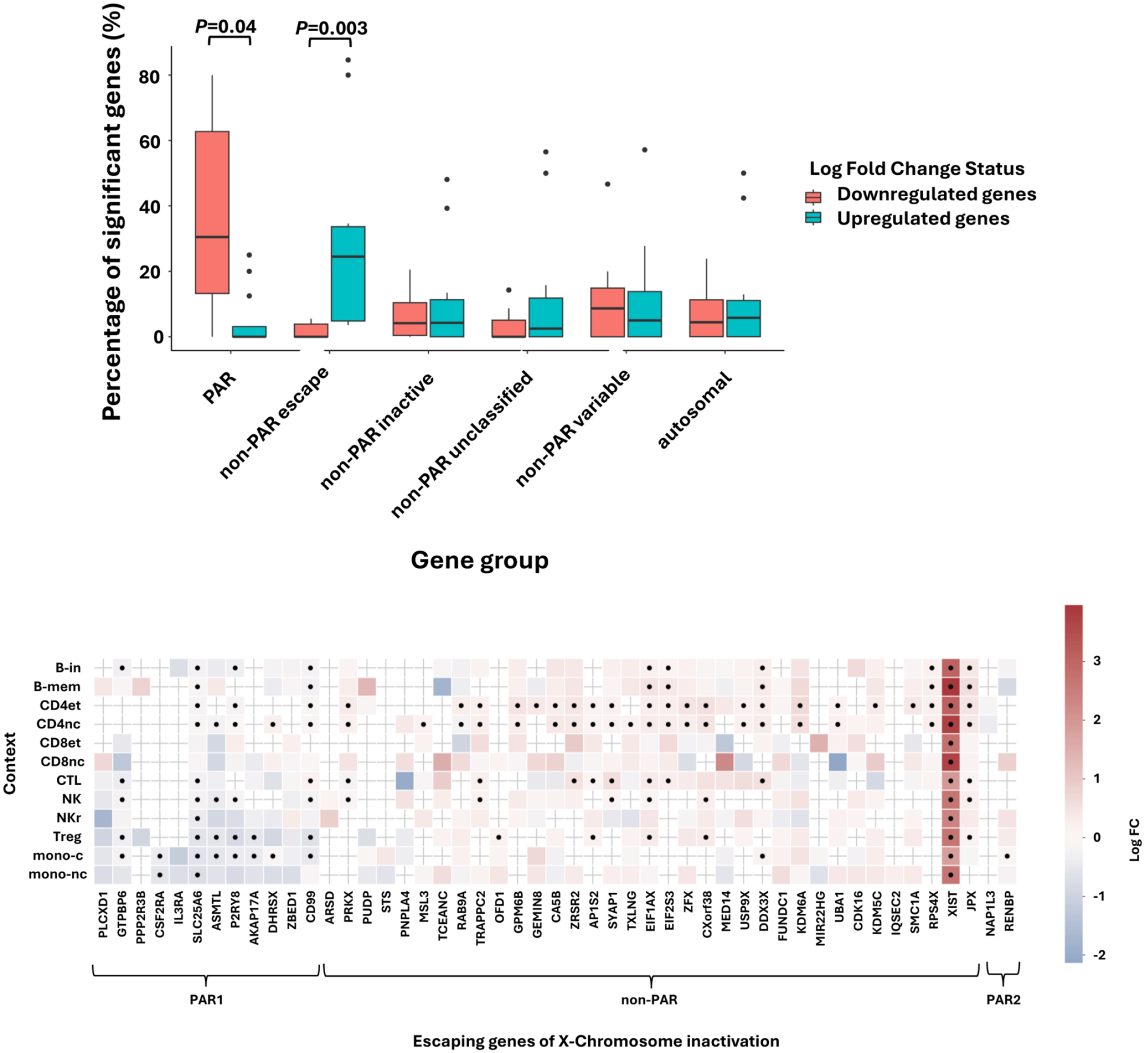
A combined representation of differentially expressed genes between LOY and non‐LOY cells. (a) Box plot showing the percentage of significantly differentially expressed genes in each cell type (*n* = 12). Genes are categorized based on their chromosomal origin: autosomal genes, X chromosome genes in the PAR region (both PAR1 and PR2), and X chromosome genes in the non‐PAR region. The non‐PAR genes are further classified as escape, inactive, variable, or unclassified following Tukiainen et al. (2017). Outliers are represented as black dots. (b) Heatmap displaying differentially expressed genes across 12 cell types, and focused on escape XCI genes. Colors represent log fold change (logFC), and significant results (adjusted *p* value < 0.05) are marked with dots.

A recent study had detected an aberrant overexpression of *XIST*, a long noncoding RNA located on the X chromosome, in male cancers (Sadagopan et al. 2022). *XIST* is essential for X chromosome inactivation (XCI) and is normally expressed in female cells. We observed *XIST* overexpression in LOY cells across all cell types. This pattern was further supported by a bimodal distribution (Hartigan's dip test, *p* < 1 × 10^−16^, Figure S3) of *XIST* expression in LOY cells. XIST expression was consistently higher in LOY cells across multiple cell types. In mono‐c, *XIST* was detected in 0.06% of LOY cells compared with 0.02% of non‐LOY cells, while in effector CD4 T cells (CD4et), it was detected in 0.23% versus 0.04%, respectively (Table S7). Overall, the most pronounced differences were observed in CD4et and CD4nc cells, with 0.13% of LOY cells expressing *XIST* compared with 0.02% of non‐LOY cells (*p* < 1 × 10^−300^), as presented in Table S5 and Figure S2. The proportion pseudobulk analysis for *XIST* showed a consistent direction of effect across all cell types (Table S8). To assess the potential XCI features associated with LOY, we classified X‐chromosome genes in our results analysis, following a previously curated list of the inactivation degree derived from GTEx data, including whole blood samples (Tukiainen et al. 2017), into inactive, escape (genes that escape XCI mechanism) and variable, and according to locus into PAR, and non‐PAR. Within 99 genes classified as escape in non‐PAR genes, we identified 26 genes (*XIST* + 25 genes) with DGE *p*_adj_ < 0.05 in at least one cell type. The majority of these genes (23 out of 26) were significantly upregulated, with no cell type showing downregulation, whereas two genes showed both upregulation and downregulation, and one gene was only downregulated (Figure 3a, Table S5). Within each cell type, on average, 37% of escape genes are upregulated, compared with 4.8% downregulated (Wilcox rank test, *p* = 0.04; results detailed in Table S9).

To eliminate the possibility of contamination with female cells, we repeated the analysis after excluding cells that express XIST (*n* = 8764 cells), which represent 0.036% of cells used in the previous differential analysis (Table S10) and observed a similar trend that 19 out of 25 escape genes were significantly upregulated in all cell types. The 19 escape genes (Figure 3b) include *KDM6A*, *DDX3X*, and *KDM5C*. These three genes have been identified to harbor more loss‐of‐function mutations in male cancer samples in comparison to female samples in the TCGA cohort (Dunford et al. 2017). A similar male bias was identified in *ZRSR2*, a splicing gene associated with myeloid malignancies, and upregulated in T cells with LOY (Togami et al. 2022). The 19 genes also include *JPX*, a long noncoding regulator for XCI, and *RPS4X*, a ribosomal gene that is overexpressed in transformed myelodysplastic syndrome (Sridhar et al. 2009). *EIF1AX*, X‐linked homolog of *EIF1AY*, was identified in addition to *DDX3X* as the top gene expression dependency for the LOY cell lines in Cancer Cell Line Encyclopedia (CCLE) (Qi et al. 2023). On the other hand, only 23% of the inactive genes (*n* = 143/431 genes) showed significant DGE in at least one cell type with a balanced distribution of association direction between upregulated genes (*n* = 74) and downregulated genes (*n* = 43) in all cell types. Finally, nine X‐PAR genes showed significant DGE between LOY and non‐LOY (*p* = 2.9 × 10^−3^), six genes were downregulated with non‐cell type upregulated (*P2RY8*, *SLC25A6*, *GTPBP6*, *ASMTL*, *CSF2RA*, and *AKAP17A*).

To assess the effect of age on the identified DGE signals, we compared gene expression profiles between older women (aged ≥ 67, *n* = 283) and younger women (*n* = 282) in OneK1K within each cell type. Unlike previous observations, we did not observe a consistent trend of upregulation among escape genes located in the non‐PAR region. Of the 19 escape genes associated with LOY in men, 16 genes were downregulated or did not show significant change in older women that includes XIST and genes connected to cancer such as *KDM6A*, *DDX3X*, and *KDM5C* (Table S11). As it was not observed in elderly women, this result suggests that the upregulation of X‐inactivation escape genes is specific to LOY rather than a general effect of aging.

To expand on these observations, we explored the results from a recent study that screened cell lines to detect gene dependencies of LOY cell lines in comparison to normal cell lines (Qi et al. 2023). Sixty out of 17,385 genes showed significant differences (*q* value < 0.05) between LOY and wild type (WT) cell lines from the Cancer Cell‐line Encyclopedia (Barretina et al. 2012). Of these, four escape XCI genes located on non‐PAR (*RPS4X*, *DDX3X*, *ZFX*, and *EIF1AX*) have the largest difference in average expression between LOY and WT cell lines. These results collectively show XCI characteristics associated with LOY, in particular, the upregulation of genes that escape XCI. These genes are known for sex bias in healthy tissue (Tukiainen et al. 2017), as well as in hematological and non‐hematological cancers (Sadagopan et al. 2022).

### 2.4 The Dynamic Effect of LOY on Cells

To further understand the way that LOY affect immune cell fate, we carried out two orthogonal analyses. (i) We conducted gene overrepresentation analysis for LOY‐associated genes, and used hallmark gene sets from MSigDB (Subramanian et al. 2005). Genes were selected if they had a logFC > 0.25 for upregulated genes, and < −0.25 for downregulated genes, and the analysis was restricted to cell types with the largest representation of LOY for each lineage (full results are presented in Table S12). (ii) We assessed the effect of LOY on gene expression across five pseudotime trajectories of cell transition from B‐in to B‐mem, mono‐c to mono‐nc, NK to NKr, CD8nc to CD8et, and CD4nc to Treg. The analysis was restricted to genes that expressed in at least three of six quantiles using both a linear and quadratic model, and adjusting the analysis for age, and 10 PCs (Yazar et al. 2022). Full dynamic analysis results are presented in Table S13. These two approaches provide a functional view of the dynamic effect of LOY across different cell types.

**FIGURE 4:**
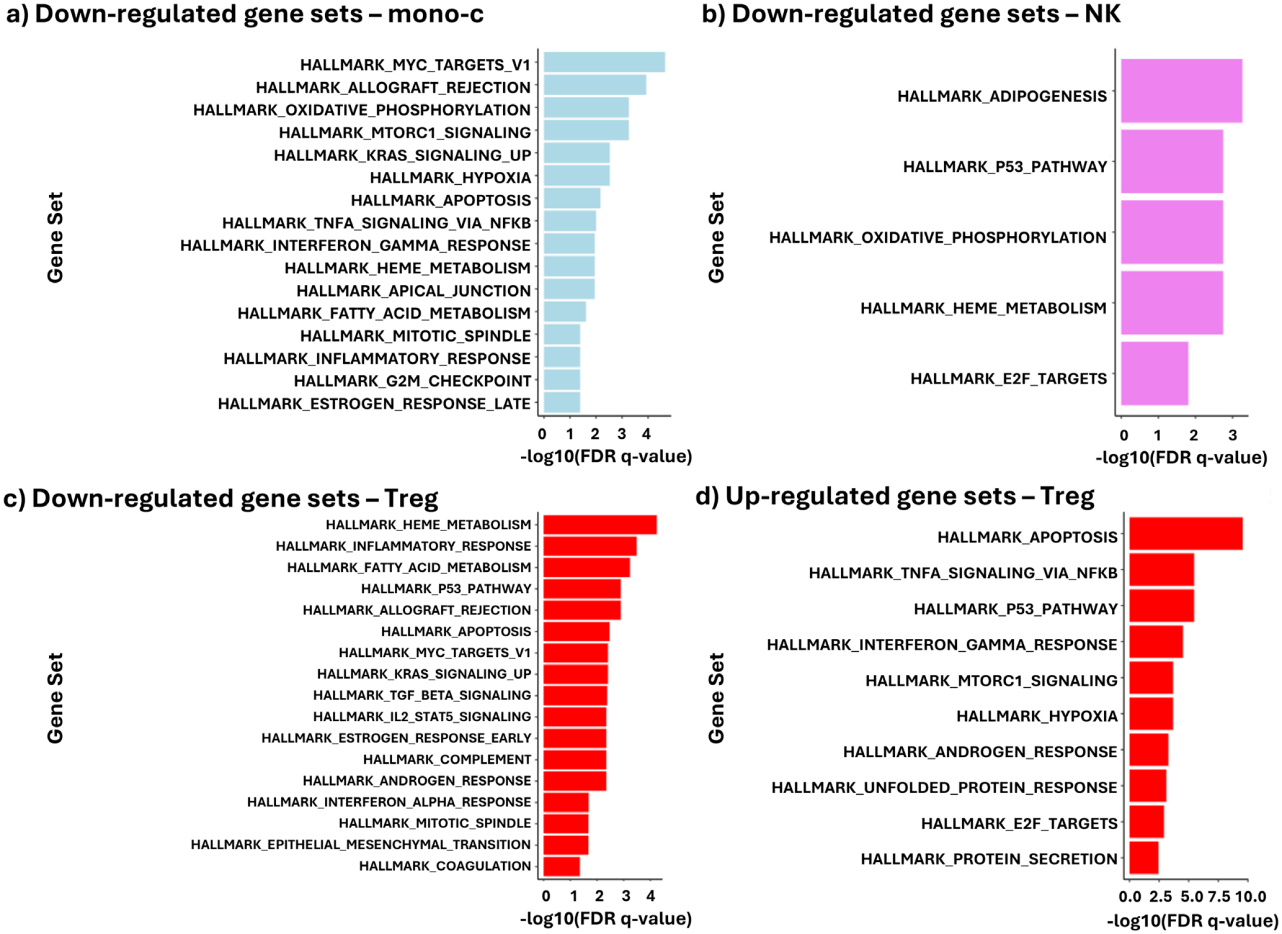
Gene overrepresentation results using molecular signature database (MSigDB), and focusing on significantly (*p* < 0.05) differentially expressed genes between LOY and non‐LOY cells. Boxplots are shown for cell subtypes with the highest LOY frequencies within each lineage: classical monocytes, regulatory T cells (Treg), and natural killer (NK) cells. Full results are provided in Table S12. This analysis was performed after excluding XIST‐expressing cells. Panels show results for (a) downregulated genes with log FC < −0.25 in classic monocytes, (b) downregulated genes with log FC < −0.25 in NK cells, (c) downregulated genes with log FC < −0.25 in Treg, and (d) upregulated genes with log FC > 0.25 in Treg.

Over the monocyte trajectory, LOY decreased the expression of nonclassical monocyte markers *LYPD2* (linear model, interaction estimate = −0.08, *p*_fdr_ = 3.73 × 10^−8^), and *C1QA* (linear model, interaction estimate = −0.07, *p*_fdr_ = 5.65 × 10^−5^), and an increase in the classical monocyte marker *RNASE2* (linear model, interaction estimate = 0.06, *p*_fdr_ = 7.9 × 10^−3^), confirming our previous lineage bias finding (Wong et al. 2011). Interestingly, the highest impact of the interaction between LOY and pseudo time was achieved on the *IGFBP6* gene, an IGF binding‐protein regulator, which increases monocyte migration from blood to tissue (Liso et al. 2017). Furthermore, the gene overrepresentation analysis of mono‐c highlighted *MYC* transcriptional factor targets as the most significant (genes overlap ratio = 0.05, *p*_fdr_ = 2.22 × 10^−5^) pathway for the downregulated genes (Figure 4a). However, this did not include a significant change in *MYC* gene expression, but we observed downregulation of *MYC* paralog: *MYCL* (log FC = −0.55, *p*_fdr_ = 3.00 × 10^−6^) exclusively in mono‐c. Interestingly, *IL1B* was significantly downregulated (log FC = −0.22, *p*_fdr_ = 2.84 × 10^−6^) in mono‐c cells with LOY, but not in any other cell type. Using a proportion‐based pseudobulk approach, calculating the fraction of *IL1B* expressing cells per donor, mono‐c with LOY showed a lower proportion compared with non‐LOY mono‐c (0.17 vs. 0.22; Beta = −0.06; *p*_fdr_ = 3.90 × 10^−3^), confirming that the observed *IL1B* downregulation is robust at the donor level.

On the other hand, older women (aged ≥ 67) showed upregulation (log FC = 0.38, *p*_fdr_ = 1.36 × 10^−3^) of *IL1B* in mon‐c compared with younger women (aged < 67), suggesting that the observed downregulation of *IL1B* in males is not driven by age‐related effects. *IL1B* is a marker of inflammation, positively associated with aging (Roubenoff et al. 1998), and its downregulation in macrophages suggests a bias toward fibrotic macrophages rather than inflammatory (Sano et al. 2022). Pathway analysis of the downregulated genes in monocytes indicated multiple signals connected to *IL1B*, including genes linked to NF‐κB (*p*_fdr_ = 0.01), KRAS signaling (*p*_fdr_ = 2.97 × 10^−3^), and inflammation response (*p*_fdr_ = 0.04). Consistently, prior analyses of serum proteins in the UK Biobank have shown downregulation of *IL1R1* and *IL1R2* and upregulation in individuals with LOY (Weyrich et al. 2025). Further supporting this pattern, *LGALS2* was downregulated (log FC = −0.16, *p*_fdr_ = 5.15 × 10^−11^) in mono‐c cells with LOY. *LGALS2* encodes galectin‐2, a CD14 ligand that induces the pro‐inflammatory phenotype in monocytes and macrophages (Yıldırım et al. 2015). Additionally, *CD86* (log FC = −0.30, *p*_fdr_ = 1.56 × 10^−4^) and MHC class II genes (*HLA‐DRA*, *HLA‐DRB1*, *HLA‐DRB5*), which are critical for antigen presentation and T cell co‐stimulation, were significantly downregulated in classical monocytes with LOY (Table S5). This pattern indicates that LOY monocytes may exhibit impaired antigen‐presenting capacity and a shift away from pro‐inflammatory activation toward a more immunosuppressive or profibrotic state (Monaco et al. 2019). Together, these findings indicate a broad attenuation of pro‐inflammatory programs in classical monocytes with LOY.

NK cells show a significant decrease in *XCL1* associated with the LOY and the transition toward recruiting Natural killer cells (NKr). *XCL1* is a chemokine that is produced in activated NK cells, which supports our previous observation that LOY attenuates NK activation to NKr. The downregulated genes associated with LOY are notably enriched in heme metabolism (*p*_fdr_ = 1.79 × 10^−3^) and adipogenesis markers (*p*_fdr_ = 5.45 × 10^−4^) (Figure 4b). These observations suggest that LOY may attenuate NK cell activation, potentially leading to alterations in NK function, particularly in the context of inflammation and metabolism.

In the trajectory of developing Treg cells, *FOXP3* and *RTKN* have the most significant positive relationship with LOY; both are known markers for Treg cells (Table S13). On the other hand, *ANXA1* is the most significantly downregulated gene. Gene overrepresentation analysis of downregulated genes in Treg cells with LOY revealed strong representation of heme metabolism (*p*_fdr_ = 5.74 × 10^−5^) and inflammation response (*p*_fdr_ = 3.26 × 10^−4^) pathways (Figure 4c), whereas upregulated genes were significantly enriched for apoptosis (*p*_fdr_ = 2.71 × 10^−10^), p53 signaling (*p*_fdr_ = 3.46 × 10^−6^), and the TNFα signaling via NF‐κB (*p*_fdr_ = 3.46 × 10^−6^) pathway (Figure 4d). These results confirm previous observations that LOY polarizes CD4^+^ T cells toward a Treg state and induces an abnormal function (Mattisson et al. 2024).

Finally, LOY exhibits its strongest effect in B cells by the downregulation of *HLA‐A*, which reflects the bias toward B‐in. *HLA‐A* has previously been connected to the risk of LOY in the UK Biobank through GWAS (Thompson et al. 2019). However, overrepresentation analysis did not identify any significantly associated hallmark gene sets in B cells or CD8^+^ T cells.

These results collectively suggest that LOY alters the fate of immune cells, as exemplified by LOY monocytes being driven toward classic monocyte phenotypes with high affinity for tissue migration and transformation into fibrotic macrophages.

## 3 Discussion

Age‐related somatic LOY in leukocytes is known to have a wide variation in prevalence across cell types and is connected to different age‐related diseases (Dumanski et al. 2021; Mattisson et al. 2024; Wang et al. 2024). Here, we identified lineage‐specific biases across the developmental trajectories of the major cell types. Notably, we found a high prevalence of LOY in mono‐c cells, resulting in expression profiles that suggest a transition toward fibrotic, rather than inflammatory, macrophages. Furthermore, we expanded the previous knowledge regarding the molecular effect of LOY (Mattisson et al. 2021), on autosomal genes (Dumanski et al. 2021) and methylation (Wright et al. 2017), and included XCI characteristics, mainly upregulation of escape XCI genes, and aberrant expression of the long non coding *XIST*.

Our analysis confirms that SNP arrays underrepresent several immune cell types, whereas scRNA‐seq enables a balanced assessment of both the myeloid and lymphoid compartments. Using scRNA‐seq, we captured a continuous drop in LOY cells across the trajectory of developing B cells, NK, and monocytes, and an increase in LOY along the developmental trajectory of Treg and CD8^+^ T cells. These trends expand on the previously observed overrepresentation of LOY in monocytes, NK cells, and Treg. Variations across different cell types may be attributed, in part, to differences in cell lifespan. For instance, B cells have varying lifespans, from weeks for naïve B cells to years for memory B cells, and even longer for CD4^+^ and CD8^+^ T cells. However, the shorter lifespans of monocytes (1–2 days) indicate a drastic impact of LOY on cell fate, which the cell lifespan cannot explain. An alternative explanation is that LOY may be associated with selective retention or impaired transition of monocytes in the classical state, rather than directly accelerating differentiation along the trajectory. This interpretation is consistent with our observation of reduced expression of nonclassical markers such as *LYPD2* and *C1QA*, as well as the profibrotic transcriptional signature observed in LOY monocytes.

Additionally, while we used pseudotime‐based approach in this study, RNA velocity analysis was not applied. Because RNA velocity infers incorrect dynamics in steady‐state human hematopoiesis due to violated model assumptions, as previously described (Barile et al. 2021; Bergen et al. 2020; Gorin et al. 2022). Experimental validation using functional assays and lineage‐specific perturbation models will be critical to determine whether LOY causally contributes to or merely correlates with these immune phenotypes.

Interestingly, the bias of LOY in B cells toward B‐in marked with *TCL1A*, rather than B‐mem marked with *CD27*, suggests an explanation for the previous finding of overexpression of *TCL1A* in B cells (Thompson et al. 2019). Our findings show that the overexpression of *TCL1A* in LOY cells is associated with attenuation of the transition from B‐in to B‐mem. This supports the previous finding that *TCL1A's* effect on clonal expansion is derived from its aberrant expression in hematopoietic stem cells (Weinstock et al. 2023). rs2887399 near *TCL1A* was the first SNP to be connected to the risk of LOY (Terao et al. 2019; Thompson et al. 2019; Wright et al. 2017; Zhou et al. 2016). The bias of LOY toward TCL1A^+^ CD27^−^ B‐in cells provides a potential fine‐mapping for rs10849448, which has previously been linked to the risk of LOY (Terao et al. 2019; Thompson et al. 2019). This SNP is located in *LTBR* (12p13.31), and near *CD27*. While GWAS identified the association of the G allele with a higher risk of LOY, the study of eQTLs in blood connected it to the downregulation of *CD27* (Võsa et al. 2021).

Remarkably, the bias of LOY monocytes toward a classical type with downregulation of pro‐inflammatory cytokine, *IL1B*, indicates a possible prestate for migration of LOY monocytes toward tissue and their transformation to fibrotic macrophages (Choi et al. 2023). This observation expanded the previous finding that LOY induces the polarization of heart macrophages toward fibrotic macrophages (which lack *IL1B*) rather than inflammatory macrophages (Sano et al. 2022). Future studies could investigate how LOY impacts intercellular communication networks via cytokine‐mediated signaling to better understand these downstream effects.

In addition, we showed, for the first time, a distinctive pattern of upregulation of escape XCI genes compared with other X‐chromosome genes, and we reported aberrant expression of the long noncoding RNA *XIST*. These patterns had been identified in male cancer samples, both germ and non‐germ lineages (Sadagopan et al. 2022). Our findings support previous observations of the high dependency of LOY cell lines on a set of escape XCI genes with paralogues on the Y chromosome (*RPS4X*, *DDX3X*, *ZFX*, and *EIF1AX*). This phenomenon could be explained by a compensatory mechanism that protects the cell from LOY complications. While a small proportion of cells from female donors in our study showed MSY mapped reads without association with age (*p* = 0.12), this indicated the possibility of false negatives in our LOY calls, but we prioritized specificity over sensitivity in our approach, meaning that we aimed to minimize false‐positive LOY calls even at the cost of missing some true LOY cells. Consequently, any cell with even a single MSY read, potentially due to ambient RNA contamination, was not classified as LOY. Furthermore, the exclusion of cells with *XIST* expression did not affect the results.

These results suggest a wider association between LOY and immune cells' fate, triggering abnormal functions and mediating the effect on disease pathogenesis by (i) in the myeloid compartment, LOY is associated with the transition of classical monocytes toward a profibrotic phenotype, (ii) in the lymphoid compartment, LOY attenuates the activation of NK cells, and the transformation of B cells to B‐mem, whereas it polarizes T cells toward Treg, (iii) LOY is associated with aberrant expression of XCI‐escape genes and XCI regulator *XIST*. Collectively, these insights position LOY as a strong correlate of cell type–specific immune perturbations, providing a compelling new framework to understand how age‐related genomic instability shapes immune dysfunction and disease risk in clonal hematopoiesis, opening promising avenues for targeted diagnostics and therapeutic intervention.

## 4 Methods

### 4.1 Study Cohort

The study was approved by the Faculty Ethics Committee (Faculty of Environmental and Lifesiences at University of Southampton) submission ID: 97597 “Dissecting the somatic clonal expansion using single‐cell multi‐omics technologies.” Additionally, the OneK1K cohort was approved by the Tasmania Health and Medical Human Research Ethics Committee (H0012902) and all donors gave informed consent (Yazar et al. 2022).

OneK1K is a cohort of individuals living or attending medical facilities in Hobart, Australia, and described in details elsewhere (Yazar et al. 2022). In this study, we focused on donors (*n* = 981) for whom both scRNA‐seq and genome‐wide SNP arrays data are available. All donors in this subset are identified as healthy with male (*n* = 416), representing 42% of the cohort (Table 2), and age range between 19 and 97 years (median = 68) for males and range between 19 and 93 years (median = 66) for females.

**TABLE 2:** The age, sex, and cell characteristics of OneK1K cohort.

Age group	Female		Male	
	Total cells, *n*	Total donors, *n*	Total cells, *n*	Total donors, *n*
≤ 38	78,497	57	56,226	43
39–58	140,805	108	86,668	69
59–78	390,638	301	281,280	227
≥ 79	120,844	99	93,238	77
Total	730,784	565	517,412	416

### 4.2 Alignment and Counting of Single‐Cell RNA‐Seq Data

We obtained raw and multiplexed FASTQ files from Sequence Read Archive (SRA) for runs (SRR18028378–SRR18029877) by using fastq‐dump tool from NCBI SRA‐tools. Next, we grouped FASTQ files according to analysis pools (*n* = 75) and processed each pool independently using the default parameters of Cell Ranger (v8.0.1) (Zheng et al. 2017), and GRCh38 reference release provided by Cell Ranger (refdata‐gex‐GRCh38‐2024‐A, March13, 2024). Cell Ranger generated a BAM file, and gene cell matrix for each pool. Next, the generated pool‐level BAM files and gene cell matrices (counts) were demultiplexed using subset‐bam (v1.1.0, https://github.com/10XGenomics/subset‐bam) and Seurat subset function (Butler et al. 2018), respectively, into donor‐level files by following the published individual donor barcodes file obtained from Gene Expression Omnibus (GEO) under ID: GSE196830. Furthermore, we processed the donor‐level BAM files using velocyto (v0.17) (La Manno et al. 2018), that count and classify reads into spliced, unspliced, and ambiguous.

### 4.3 Calling LOY in Immune Cells

We used all reads generated by both Cell Ranger and velocyto and mapped to Male‐Specific Region (MSY) of the Y chromosome (GRCh38, start: 2,781,480, end: 56,887,902) to classify cells into LOY or non‐LOY. LOY status is defined by the finding of no reads mapped to the MSY region.

### 4.4 Calling Both LOY and mCA Using SNP Array Data

We used MoChA, a BCFtools extension to call LOY and mCA using long‐phased genotypes from genomic SNP array data (Loh et al. 2018). Initially, we obtained the genotyping IDAT pairs of files (green and red) from GEO accession: GSE196829. Next, we converted IDAT into GTC files and to VCF files, sequentially using BCFtools plugin (https://github.com/freeseek/gtc2vcf), Illumina manifest files (GSA‐24v2‐0_A1.bpm and GSA‐24v2‐0_A1.csv), and the expected intensities file (GSA‐24v2‐0_A1_ClusterFile.egt). Next, the VCF file is phased independently using SHAPEIT5 and imputed using impute5. Finally, MoChA calls chromosomal alterations, and the default filters were applied to classify events into LOY, LOX, mCA (CNG, CNL, and CNN) events.

### 4.5 Identification of Major Cell Populations

To get a benefit from the quality control and normalization steps applied by the author, we obtained feature cell matrix from CellxGene in “rds” format, compatible with Seurat (v4) R package and assigned our LOY calls into it. Quality control was performed by transforming each QC metric into a standardized normal distribution (*Z*‐score), using percentile‐based mapping to a normal distribution. Cells with values below three negative *Z*‐scores were classified as outliers and removed. For mitochondrial gene expression, cells with two or more positive *Z*‐scores were additionally excluded to remove apoptotic cells. The original feature cell matrix contains 1,248,980 cells in 981 donors. We excluded a minimal number of cells (*n* = 784 cells) that had not been called in our Cell Ranger assignment, and we cannot detect LOY state of them. The excluded cells affect 373 donors by excluding 1–4 cells in each (548 donors have no change, 226 with 1 cell excluded, 85 with 2 cells, 43 with 3 cells, 19 with 4 cells excluded). The median read depth of the cells retained for downstream analysis was 2947 UMIs. For the sensitivity assessment, we applied three additional thresholds to exclude low‐coverage cells. We removed the lowest 5%, 10%, and 15% of cells based on UMI counts, corresponding to UMI cutoffs of 1221, 1456, and 1649, respectively. However, we retained all cells for the downstream analysis. To identify the major cell populations. Initially, we used the predicted cell types by the query‐reference cell map to group cells into five major groups: B cells, monocytes, NK cells, CD4^+^ T cells, and CD8^+^ T cells. Other cell types and outlier contaminated cells expressing CD34^+^ were excluded. Additionally, we used group‐specific contamination exclusion features: *CD4*, *CD3G/D/E*, and *CD8A/B* for B cells, monocytes, and NKs; whereas *CD8A/B* were used for CD4 T cells, and CD4, *CD3G/D/E* were used for CD8 T cells. Cell type prediction was conducted independently for males and females.

To achieve more purity, we analyzed each cell type independently. Initially, we log‐normalized counts of each subset with a scale factor of 10,000 and calculated the scaled expression of the most variable 500 features and adjusted for the percent of reads that map to the mitochondria genome and the sequencing pool. We clustered each subset using the Louvain algorithm and the constructed KNN graph based on the Euclidean distance of 30 PCs. Next, we assessed the differential expression of the cell type markers by comparing each cluster to other clusters and using the Wilcoxon Rank‐Sum test. Next, clusters that did not show a significant upregulation of the expected markers (Yazar et al. 2022) were excluded.

### 4.6 The Relationship Between LOY Status and Cell Subtype

We tested the relationship between LOY and cell type using a logistic regression model adjusted for age, sequencing pool, 10 PCs and multiple testing using the FDR approach (*n* = 20 tests). We applied two different settings (i) cell types were binarized into 0/1 for B‐in versus B‐mem, NK versus NKr, Treg versus other CD4^+^ T cells, CD8nc versus CD8et, and mono‐c versus mono‐nc, (ii) cells were classified according to their position on the pseudotime trajectory and classified into six equal quantiles. In detail, we generated a low‐dimensional embedding using the potential of heat diffusion for affinity‐based transition embedding (PHATE), the previously scaled expression data of 500 highly variable genes, and 10 PCs (Moon et al. 2019). Next, we calculated the pseudo time using slingshot (Street et al. 2018) and PHATE embeddings. We specified the start and end cluster based on biological knowledge judgment. Next, we split the cells into six quantiles across the slingshot pseudotime curve. The frequency of LOY in each quantile was calculated and presented in percentages.

For each of the two settings (binary cell type/pseudotime quantiles), we built three models (i) univariate model; (ii) multivariate model with age, sequencing pool, and 10 PCs; (iii) multivariate model using all the previous covariates in addition to rs2887399 (TCL1A). rs2887399 was encoded in an additive model (G/G = 0, G/T = 1, T/T = 2). The *p* values were adjusted for multiple testing using the FDR approach (*n* = 30 tests).

For sensitivity analysis, both the univariate and multivariate models were reestimated independently within two age strata, defined by the median age of the cohort (< 68 and ≥ 68 years), using the same model specifications as in the primary analysis.

### 4.7 Differential Gene Expression

We tested the differential expression between LOY and non‐LOY cells in males using the MAST algorithm, a two‐part hurdle model that was developed to deal with the highly frequent dropout and the bimodal distribution of the single cell expression data (Finak et al. 2015). The algorithm was applied to the log‐normalized data and used the default parameters in Seurat (v5), restricting the analysis to genes expressed in at least 1% of the cells within each group and requiring a minimum log fold change (LogFC) of 0.1 between clusters. *p* values were adjusted for multiple testing using the Bonferroni approach (*n* = 11,940). The differential gene expression analysis was replicated after excluding cells expressing XIST RNA in males and multiple testing using the Bonferroni approach (*n* = 13,922 tests). Additionally, we applied the same method to compare gene expression between older (≥ 67 years, *n* = 283) and younger (< 67 years, *n* = 282) female groups. The results were reported using average log_2_ fold change, percentage of cells where the gene is detected in, and Bonferroni‐adjusted *p* value (*n* = 3938 tests).

### 4.8 Proportion‐Expressing Pseudobulk Differential Expression

We used a proportion‐expressing pseudobulk approach to test for differences in expression frequency between LOY and non‐LOY within each cell type. For each gene, we computed the proportion of cells expressing (expression > 0) in each (donor, cell type, LOY status) group. Each such group was treated as a pseudobulk sample, yielding one proportion per gene per donor and LOY status per cell type. Only genes expressed in at least 1% of cells in either LOY population, and with a minimum effect change of 0.1. At the donor level, we compared LOY against non‐LOY using the Wilcoxon rank‐sum test on these proportions. *p* values were corrected for multiple testing using the Bonferroni method across all tested genes and cell types in the combined analysis.

### 4.9 The Dynamic Effect of LOY on Cell Fate

To test the dynamic effect of LOY on gene expression. We adopted the method used in Yazar et al. (2022) to assess the linear and quadratic effect of the interaction between LOY and quantile, that is, the position of the cell on the trajectory. For each test, we generated two models: a basic model and an augmented model. We compared the two models using an ANOVA test. The independent variables of the basic model are LOY status, quantiles of pseudotime, age, 10 PCs and under the random effect of donor. The augmented model includes the same variables in the basic model in addition to the interaction term between LOY status and quantiles of pseudotime. The independent variables of the quadratic model are LOY status, quantiles, quantiles square, age, 10 PCs and under the random effect of donor id. The augmented model includes the same variables in the basic model in addition to two interaction terms: LOY status and quantiles interaction, and LOY status and quantiles square interaction. For each gene, we reported the results of the linear and quadratic model as interaction estimate, *p* value, and Bonferroni‐adjusted *p* value (*n* = 448 tests).

### 4.10 Gene Overrepresentation Analysis

We performed gene overrepresentation analysis (ORA) using hallmark gene sets from MSigDB to evaluate the enrichment of differentially expressed genes (DEGs). Genes were analyzed separately for each cell type, with upregulated genes (logFC > 0.25, > 0.5, and 0.75) and downregulated genes (logFC < −0.25, < −0.5, and < −0.75) assessed independently. Enrichment was assessed using the hypergeometric test (equivalent to one‐tailed Fisher's exact test, with multiple testing correction performed using the Benjamini–Hochberg false discovery rate [FDR] approach).

### 4.11 Other Statistical Methods

The relationship between the percentage of LOY cells per donor and their age was tested using a linear regression model in R. LOY percentages were logit‐transformed to account for their bounded distribution between 0% and 100%. Age, 10 PCs and pool number were added as independent variables. The correlation between LOY clonal fraction estimated from SNP arrays and that estimated from scRNA‐seq was tested using Pearson's correlation test. To compare the percentage of upregulated genes with the percentage of downregulated genes across different cell types, we categorized genes based on their chromosomal location into PAR, escape non‐PAR, and inactive non‐PAR groups. We then applied the Wilcoxon rank‐sum test and reported the results as mean, median, *p* value, and Wilcoxon statistic. To assess whether *XIST* expression followed a unimodal or multimodal distribution, we applied Hartigan's dip test for unimodality. The test was performed separately for male and female cells using normalized single‐cell expression values. To account for the high proportion of zero values typical of single‐cell RNA‐sequencing data, the analysis was conducted both on the full dataset and on the subset of cells with nonzero XIST expression. Statistical significance was evaluated using permutation‐based *p* values as implemented in the diptest package (Maechler 2013).

## Author Contributions

A.D. and O.R. designed the study. A.D. performed the data analysis. L.G. developed the Shiny app. All authors refined the analysis and contributed to writing the paper.

## Funding

This work was supported by Biotechnology and Biological Sciences Research Council (BB/Y513003/1).

## Conflicts of Interest

The authors declare no conflicts of interest.

## Supporting information


**Figure S1:** The percentage of LOY across four cell trajectories. Cells were categorized into five major cell types and further classified along their developmental trajectory using pseudotime analysis, and six equal quantiles. (a, c, e, and g) Quantile projections visualized using PHATE dimensionality reduction. (b, d, f, h, and j) cell type projections visualized using PHATE dimensionality reduction. (a, b) Transition from immature and naïve B cells to B memory cells. (c, d) Transition from natural killer (NK) cells to NK recruiting cells. (e, f) Transition from CD8^+^ naïve and central memory T cells to CD8^+^ effector memory T cells. (g, h) Transition from CD4^+^ naïve and central memory T cells to CD4^+^ effector memory and central memory T cells.
**Figure S2:** The distribution of XIST gene expression in males (a) and females (b).
**Figure S3:** The distribution of XIST gene expression in male LOY (a) and male non‐LOY (b).

**Table S1:** The count and percentage of LOY cells in Tenk10K (phase 1, *n* = 981) identified from scRNA‐seq, and mosaic chromosomal alteration calls detected by SNParrays.
**Table S2:** The relationship between clonal fraction stemated from DNA genotyping by SNP arrys (whole blood), and clonal fraction estimated from scRNA‐seq (PBMCs) per cell type.
**Table S3:** The relationship between LOY and cells on the development trajectory, classified by cell subtype or pseudotime representing quantile (*n* = 6). We used logistic regression model adjusted for age, batch, 10PCs, and multiple testing. rs2887399 was encoded in a *n* additive model 0/1/2.
**Table S4:** Age‐stratified analysis for the relationship between LOY and cells on the development trajectory, classified by cell subtype or pseudotime representing quantile (*n* = 6). We used logistic regression model adjusted for age, batch, 10PCs, and multiple testing. Individuals were split by the median age (68 years).
**Table S5:** The differential gene expression of LOY cells against non‐LOY cells in 12 cells subtype.
**Table S6:** The proportion‐based pseudobulk differential expression of LOY cells against non‐LOY cells.
**Table S7:** The distribution of XIST expression in male cells.
**Table S8:** The proportion‐based pseudobulk differential expression of XIST for LOY cells against non‐LOY cells.
**Table S9:** Results of the comparison between the percentage of upregulated and the percentage of dowenregulated genes within each cell type and across six different gene classes.
**Table S10:** Differential gene expression between LOY cells against non‐LOY cells in 12 cells subtypes after excluding XIST‐expressing cells.
**Table S11:** Differential gene expression between older women against younger women cells in 12 cells subtypes after excluding XIST‐expressing cells.
**Table S12:** Gene Set Enrichment Analysis (GSEA) using hallmark of gene sets of MSigDB. Enrichment analysis were conducted using different cutoff for logFC.
**Table S13:** The dynamic effect of LOY on the cells trajectory.

## Data Availability

The single‐cell raw sequencing data were obtained from SRA for runs (SRR18028378–SRR18029877). The metadata and the genome variation profiling by SNP array were obtained from NCBI's Gene Expression Omnibus (GEO) under ID: GSE196735, GSE196830, and GSE196829. scRNA‐seq counts were obtained from https://cellxgene.cziscience.com/datasets. LOY calls, cell clusters, and pseudotime inference can be visualized by the shiny app. The app URL is available in https://github.com/ad2n15/LOY_OneK1K_Shiny. The programmatic code used to generate the results was deposited in https://github.com/ad2n15/LOY_Onek1k.
