# Supplementary material for: Loss of Chromosome Y Associates With Altered Immune Cell Trajectories and X‐Inactivation Features
Source: Aging Cell. 2026 May 3;25(5):e70528. doi: 10.1111/acel.70528 (PMC13135724; doi:10.1111/acel.70528)
Supplement: Supplementary file 1 — Figure S1: The percentage of LOY across four cell trajectories. Cells were categorized into five major cell types and further classified along their developmental trajectory using pseudotime analysis, and six equal quantiles. (a, c, e, and g) Quantile projections visualized using PHATE dimensionality reduction. (b, d, f, h, and j) cell type projections visualized using PHATE dimensionality reduction. (a, b) Transition from immature and naïve B cells to B memory cells. (c, d) Transition from natural killer (NK) cells to NK recruiting cells. (e, f) Transition from CD8+ naïve and central memory T cells to CD8+ effector memory T cells. (g, h) Transition from CD4+ naïve and central memory T cells to CD4+ effector memory and central memory T cells. Figure S2: The distribution of XIST gene expression in males (a) and females (b). Figure S3: The distribution of XIST gene expression in male LOY (a) and male non‐LOY (b). [file ACEL-25-e70528-s001.docx]

**Loss of chromosome Y associates with altered immune cell trajectories and X-inactivation features**

Ahmed Dawoud^1*^, Luke Green^2^, Owen Rackham^1*^

1. School of Biological Sciences, University of Southampton
2. Faculty of Medicine, University of Southampton

Correspondence to: [a.dawoud@soton.ac.uk](mailto:a.dawoud@soton.ac.uk); o.j.l.rackham@soton.ac.uk

Supplementary figures:


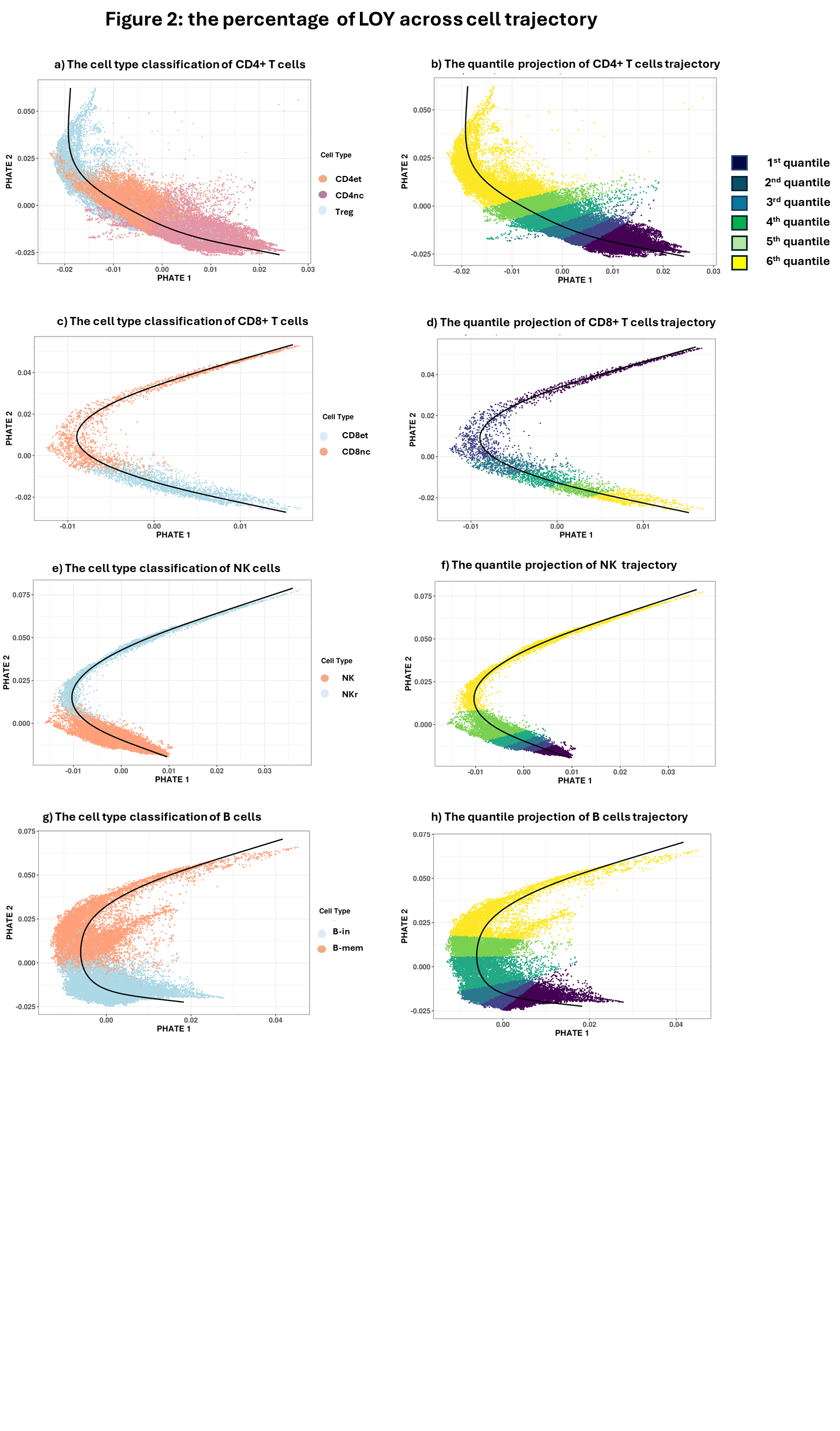


***Figure S1: The percentage of LOY across four cell trajectories. Cells were categorized into five major cell types and further classified along their developmental trajectory using pseudotime analysis, and six equal quantiles. (a,c,e,and g) Quantile projections visualized using PHATE dimensionality reduction. (b,d,f,h, and j) cell type projections visualized using PHATE dimensionality reduction. (a,b) Transition from immature and naïve B cells to B memory cells. (c,d) Transition from natural killer (NK) cells to NK recruiting cells. (e,f) Transition from CD8+ naïve and central memory T cells to CD8+ effector memory T cells. (g,h) Transition from CD4+ naïve and central memory T cells to CD4+ effector memory and central memory T cells.***

**
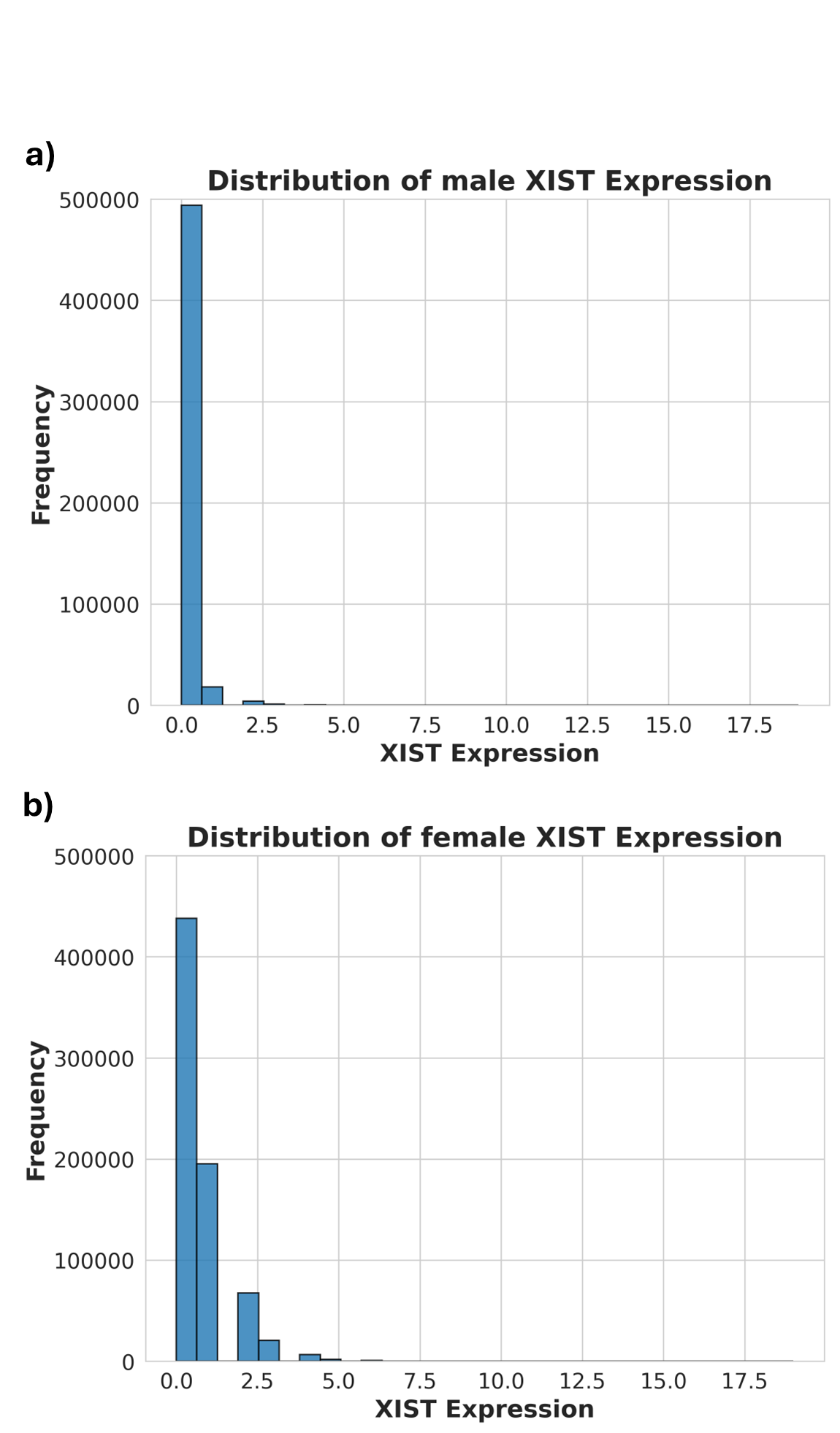
**

**Figure S2: The distribution of XIST gene expression in males (a) and females (b)**


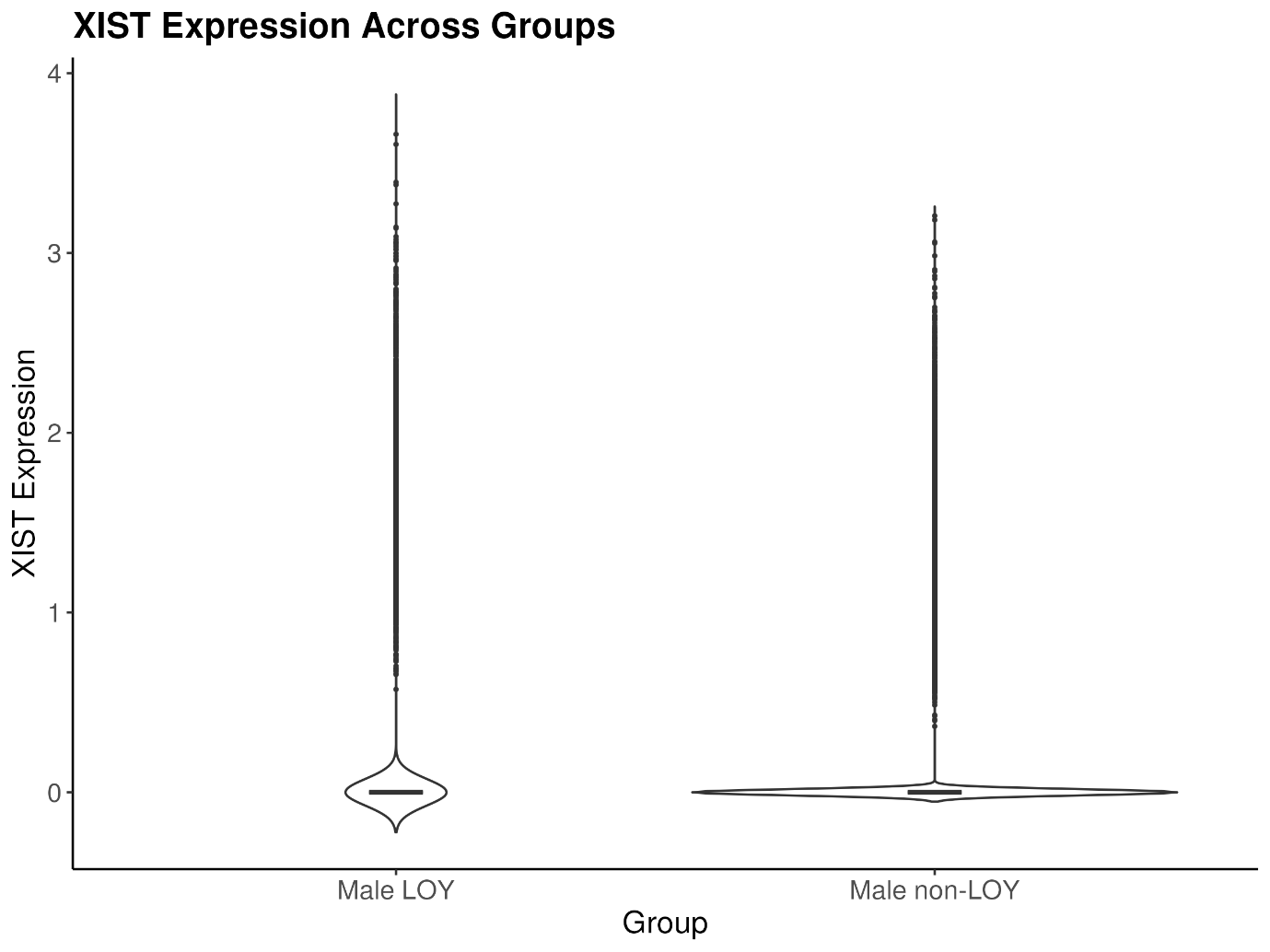


**Figure S3: The distribution of XIST gene expression in male LOY (a) and male non-LOY (b)**
